# Large euarthropod carapaces from a high latitude Cambrian (Drumian) deposit in Spain

**DOI:** 10.1098/rsos.230935

**Published:** 2023-10-25

**Authors:** Stephen Pates, Samuel Zamora

**Affiliations:** ^1^ Department of Zoology, University of Cambridge, Cambridge CB2 3EJ, UK; ^2^ Instituto Geológico y Minero de España (IGME-CSIC), 50006, Zaragoza, Spain; ^3^ Grupo Aragosaurus-IUCA, Área de Paleontología, Facultad de Ciencias, Universidad de Zaragoza, Zaragoza, Spain

**Keywords:** Euarthropoda, bivalved carapace, Iberian Chains, Mesones de Isuela, non-biomineralized fossils

## Abstract

Deposits preserving non-biomineralized tissues and animals provide an unrivalled opportunity to study the evolution and radiation of early animal life. Numerous sites of Cambrian age are known from North America (Laurentia) and South China (East Gondwana), which provide a high resolution picture of the fauna at low latitudes. By contrast, our knowledge of Cambrian animals from higher latitudes is relatively poor. This patchiness in our knowledge of animal life during the radiation of animals in the Cambrian period limits our ability to understand and detect palaeogeographic trends and does not provide a full appreciation of animal diversity at this time. Here we report a new middle Cambrian (Drumian) site preserving lightly sclerotized euarthropod carapaces, sponges and palaeoscolecids near the village of Mesones de Isuela in the Iberian Chains (Spain). We describe three bivalved euarthropod carapace morphs, two comparable to those described from the only other high latitude Drumian deposit, the Jince Formation (Czechia), and one distinct from previous discoveries. These new findings highlight the importance of high latitude Gondwana Konservat Lagerstatten for understanding the palaeogeographical aspect of the radiation of early animals and suggest that bivalved euarthropods at high latitudes were larger than those at lower latitudes during the Cambrian.

## 1. Introduction

**Figure 1.**
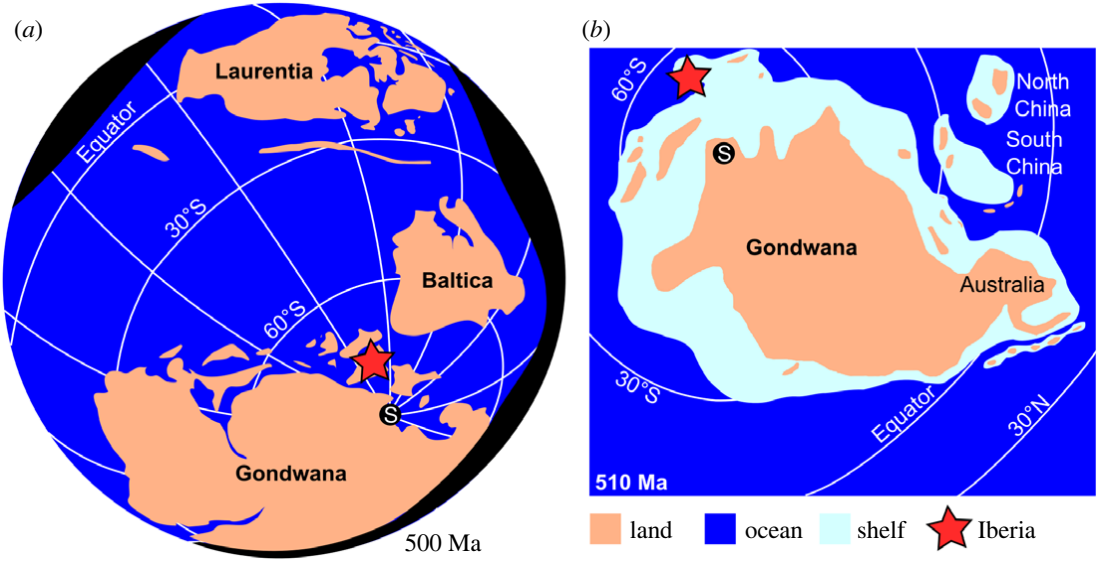
Reconstruction of palaeocontinent positions for the middle Cambrian. (*a*) Reconstruction at 500 Ma, showing position of Gondwana relative to Laurentia, Baltica and South Pole (redrawn from [16] fig. 5.3). (*b*) Reconstruction at 510 Ma, showing Gondwana and adjacent areas, including detail of ocean, shelf and land (redrawn from [16] fig. 5.4). S, South Pole. Note that a similar high latitude position for Iberia and low latitude positions for Laurentia, South China, North China and Gondwana deposits such as the Emu Bay Shale are also recovered by other workers such as ref [17].

Fossil assemblages that preserve non-biomineralized tissues provide a view of the evolution and ecology of early life not available from more abundant biomineralized skeletons [1,2]. However these exceptional deposits are not equally distributed in time or space [3–5] nor do they preserve the same tissues or with equal fidelity [2,3,6]. Data from globally distributed deposits—in both the palaeo and modern senses—are vital to more fully understand the composition and evolution of early Palaeozoic ecosystems and animals. However within the Cambrian, most deposits preserving non-biomineralized tissues can be found between 30° N and 30° S [1,7–15], on the palaeocontinent Laurentia, on the South China or North China blocks, or close to the Equator on palaeocontinent Gondwana (figure 1). Higher latitude sites of Cambrian age are rarer, known from Baltica and Gondwana [18–24]. The limited geographical distribution of deposits preserving non-biomineralized tissues is even more apparent in the Drumian, with deposits from higher latitudes only reported from Gondwana, specifically Czech Republic [18,24] (Buchava and Jince Formations) and Spain (Murero Formation within the Badules Unit, see §1.1).

Here we present a mid-high palaeolatitude new locality in Spain (figure 1), representing a high latitude site in Gondwana. This is the first from the Mesones Unit known to preserve lightly sclerotized and mineralized organisms including euarthropods, palaeoscolecids and sponges. We also describe non-biomineralized euarthropod carapaces, representing at least two, possibly three taxa, with similarities to carapaces described from other deposits from high latitudes in Gondwana, but distinct from those at lower latitudes.

### 1.1. Exceptionally preserved fossils from the Cambrian of Spain (Badules Unit)

**Figure 2.**
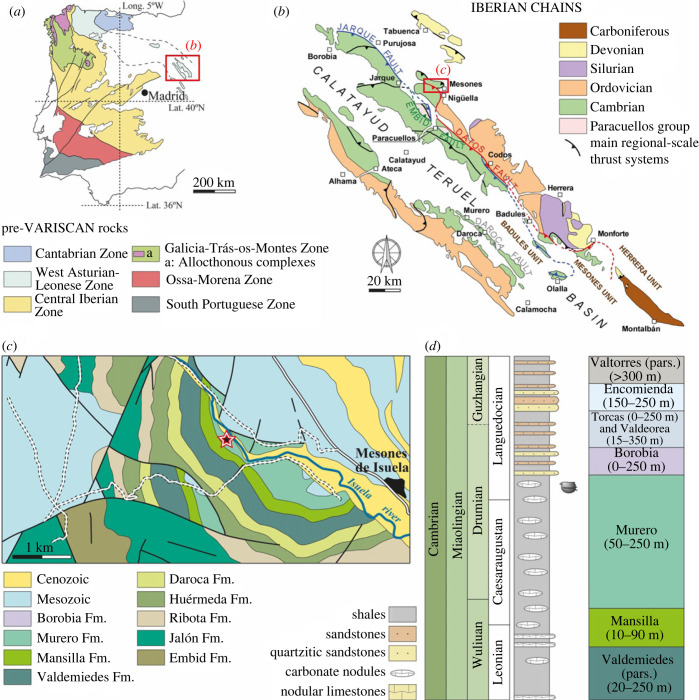
(*a*) Sketch of the Iberian Massif showing the different structural and palaeogeographic zones with Neoproterozoic and Palaeozoic rocks. The red square indicates the map detailed in (*b*) after [25]. (*b*) Geological map of the Iberian Chains with indication of Palaeozoic outcrops and tectonostratigraphic units, after [26]. Note the location of the new locality in the Mesones Unit. (*c*) Geological map of the studied area with indication of the locality in the Murero Formation (after [27]). (*d*) Simplified log of the Cambrian formations from the Iberian Chains with indication of the levels containing the new described fauna.

The Iberian Chains in northeast Spain are two large Palaeozoic outcrops (Western and Eastern Iberian Chains) trending northwest-southeast, separated by the Tertiary Calatayud-Teruel Basin and considered the central part of the Iberian Cordillera (figures 1 and 2). In this region, Palaeozoic rocks are structured into three tectonostratigraphic units separated by important faults (Jarque and Datos faults); from southwest to northeast they are named the Badules, Mesones and Herrera Units [28]. Levels with slightly sclerotized fossils from the Cambrian of Spain have previously been reported from the Murero biota in the Badules Unit, from Valdemiedes and Murero Formations. Although these levels have been considered as Burgess Shale-type deposits by some previous authors [29], fossils from Murero correspond to slightly sclerotized organisms only and entirely non-cuticularized animals are absent, which is a great difference between Murero and other important Cambrian deposits, such as the Burgess Shale and Chenjgiang biota (for a discussion, see [6]).

Palaeoscolecids [22,30,31], algae and sponges [32–35] have been reported from the Murero Formation, and a taphonomic study from a short interval of the Murero Formation in Rambla de Valdemiede has been completed [36]. Some specimens of the sponge *Leptomitus conicus* are also known from nearby Jarque [33], a locality providing a slightly skeletonized fauna outside Murero, that also falls within the Badules Unit.

Non-biomineralized fossils have been reported from the slightly older Valdemiedes Formation thanks to relatively large excavations that were carried out by Liñán in the 1990s. These excavations provided a rich assemblage of shelly fauna and poorly mineralized taxa that were presented to the scientific community in a meeting focused on exceptional preservation [29]. The Valdemiedes Formation includes the palaeoscolecid *Schistoscolex* [37] the sponge *Crumillospongia mureroensis* [21] and the radiodont *Caryosyntrips* cf. *C. camurus* [38]. The latter was originally described as a partial body of a lobopodian [39]; however comparisons with well-preserved material from the Burgess Shale and Cambrian deposits in Utah allowed assignment of this specimen to *Caryosyntrips* [38] (though see subsequent discussion in [40,41]).

## 2. Material and methods

The new fossil material was collected from a locality near Mesones de Isuela, a small village situated 75 km westsouthwest of Zaragoza, in the north part of the Iberian Chains (figure 2). Specimens were legally sampled under permission EXP:220/2016 from the Service of Prevention, Protection and Research of the Aragón Government.

**Figure 3.**
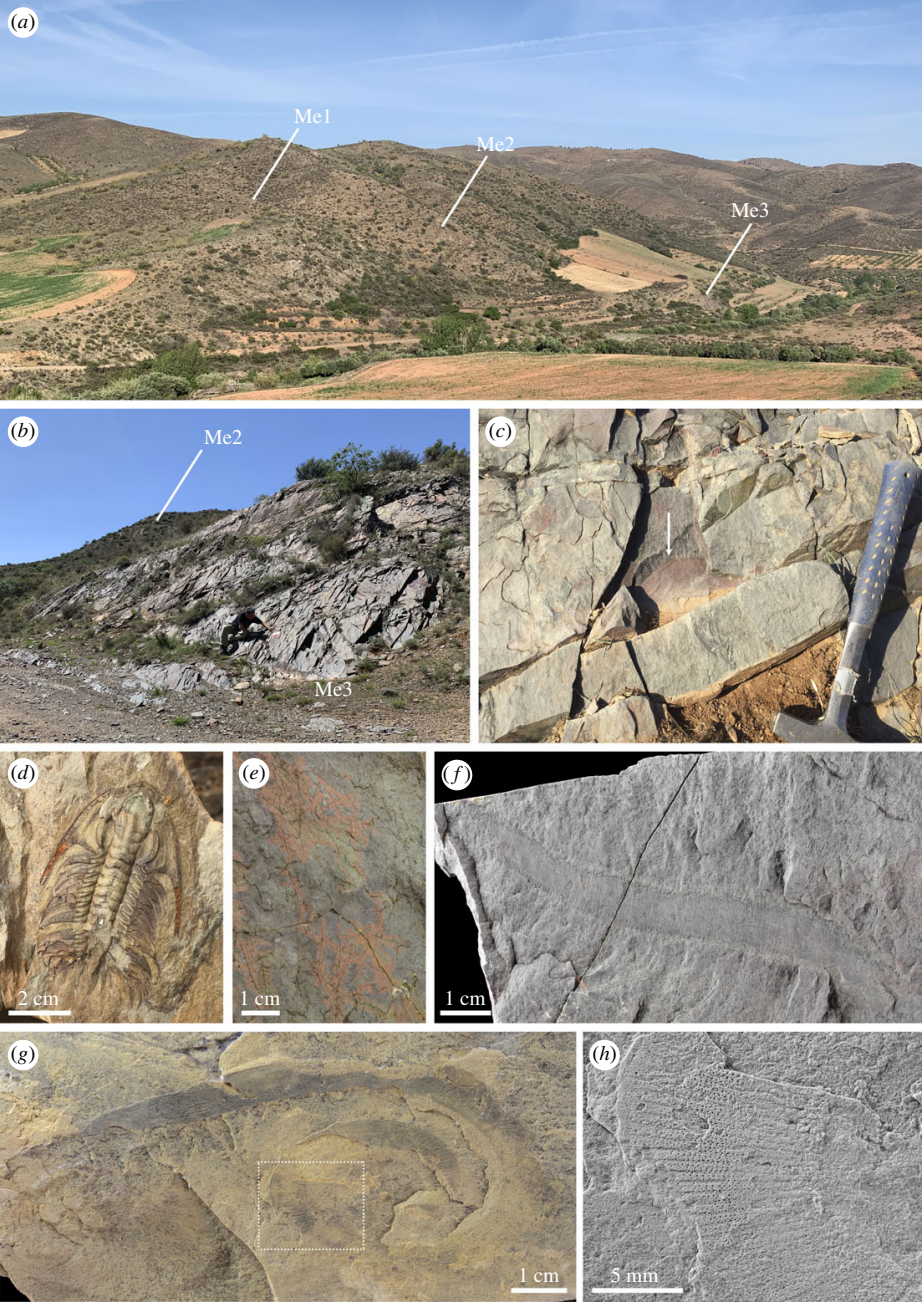
(*a*) Panoramic view of the area from the north side of the Isuela river with indication of the exact places where the fauna has been collected. (*b*) Detail of bed planes containing euarthropods, palaeoscolecids and sponges. (*c*) Specimen of euarthropod B before extraction. (*d*) Complete specimen of the trilobite *Eccaparadoxides brachyrhachis* collected from Me2. (*e*) Indeterminate reticulosan sponges from Me3. (*f*) Partial palaeosclecid *Wronascolex*? collected from Me3. (*g*) Complete palaeosclecid *Wronascolex*? collected from Me1. (*h*) Detail of previous specimens showing sclerite arrangement. Specimen figured in (*e*) is submerged in water; (*f*) and (*h*) are photographed whitened with sublimated ammonium chloride.

The sampled section belongs to the Mesones Unit [28] (figure 2*b*), and the new site is the first one in this unit to provide slightly skeletonized fossils. Valenzuela *et al*. [42] first mapped the area in detail and described the stratigraphy and fossil content. The Mesones de Isuela locality outcrops 500 m east of the M3 section [42]. The Murero Formation (figure 2*c,d*) in this area has been subdivided into two parts: a lower part with green shales alternating with sandy units, and an upper part with red shales. All specimens of euarthropods come from three different levels and have been collected from the upper section of the green shale, just below the appearance of the first sandy unit that are stratigraphically equivalent and fall within the Murero Formation. One of the levels has provided euarthropods, reticulosan sponges and one palaeoscolecid (Me3 in figure 3); the other has provided only isolated fragments of euarthropods (Me2 in figure 3). These two levels appear slightly separated but facies and faunal content suggest that they are probably equivalent. A thin level that crops about 20 m from Me2 has provided only one complete palaeoscolecid (Me1 in figure 3). Fossil assemblage includes trilobites (*Solenopleuropsis thorali*, *Solenopleuropsis marginata*, *Eccaparadoxides brachyrhachis*, *Eccaparadoxides pradoanus*, *Conocoryphe heberti*, *Peronopsis* sp.), echinoderms (*Gyrocystis platessa*, *Lichenoides* sp., isolated holdfast indet. and *Gogia* sp.) and indeterminate phosphatic brachiopods. These levels have been also studied for injuries in trilobite carapaces [27]. They appear situated within the *Solenopleuropsis thorali* biozone, which correspond to the regional lower Languedocian Stage [43] of the Mediterranean subprovince (northeast Spain). This interval correlates with the global Drumian stage [44] (figure 2*d*).

This study focuses on seven specimens of partial carapace material collected during fieldwork that began in the autumn of 2016, with additional trips in 2017 and 2023. Specimens are accessioned at Museo de Ciencias Naturales de la Universidad de Zaragoza (MPZ).

Specimens were photographed both dry and submerged in water. Some of them were whitened with ammonium chloride sublimated to enhance the ornamentation. In some cases latex peels were constructed and whitened with ammonium chloride prior to photography, to show fine details such as the ornamentation more clearly. Linear measurements were made using ImageJ [45] from specimen photographs. Line drawings and figures were constructed in Inkscape 1.1.

## 3. Results

Partial carapace material corresponding to three distinct morphologies were recognized. These can be distinguished from one another by their distinct ornamentations and outline shapes.

### 3.1. Systematic palaeontology

*Phylum* Euarthropoda Lankester 1904 [46] (*sensu* [47])

*Class* Uncertain

*Order* Uncertain

*Family* Tuzoiidae Raymond 1935 [48]

Type genus *Tuzoia* Walcott 1912 [49]

Other genus *Duplapex* Ma *et al*. 2022 [50]

*Remarks.* Recent workers have referred Tuzoiidae to the order Tuzoiida [50], Hymenocarina [51,52] or left it uncertain [53]. The most recent phylogenetic analysis [51] recovered *Tuzoia* as an early diverging total-group mandibulate, as part of a clade comprising fuxianhuiids, hymenocarines and crown mandibulate groups pancrustaceans and myriapods. However, Hymenocarina was not recovered as a monophyletic group in this analysis. Thus we leave the class and order uncertain for the family Tuzoiidae.

**Tuzoiidae indet.**

**Figure 4.**
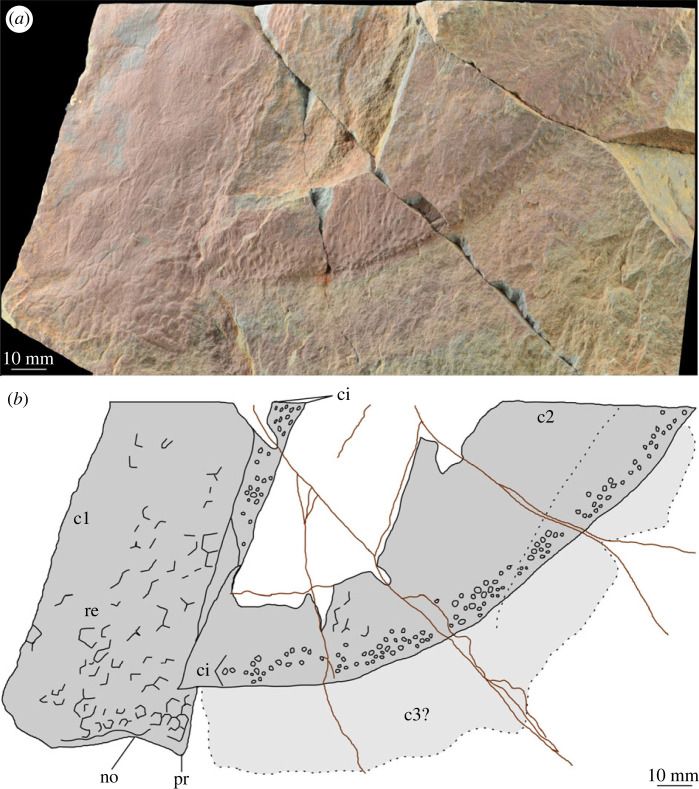
MPZ 2023/197. Partial carapaces of Tuzoiidae indet. and euarthropod A from Mesones de Isuela (Zaragoza, Spain), *Solenopleuropsis thorali* biozone, Murero Formation, preserved in external view. (*a*) Specimen photograph; (*b*) interpretative drawing. c1, c2; carapace 1 (Tuzoiidae indet.) and carapace 2 (euarthropod A); c3?, possible third carapace, pair to c2; ci, elliptical/circular indentation ornament; no, notch; pr, process; re, reticulation ornament composed of polygons.

**Figure 5.**
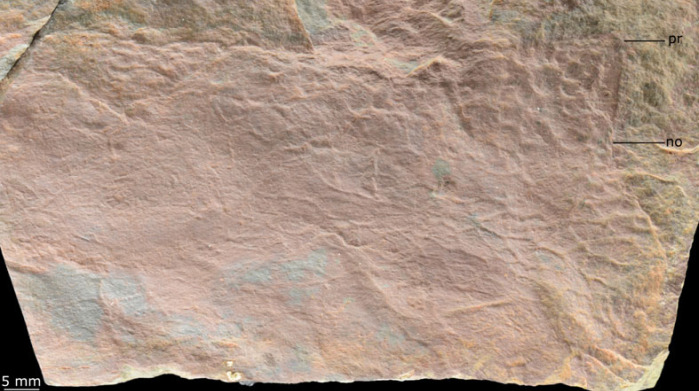
MPZ 2023/197. Partial carapace of Tuzoiid indet. (close up of c1 in figure 4, rotated so that dorsal margin of carapace is parallel to figure margin). no, notch; pr, process.

Figure 4 (c1), figure 5

*Material.* A single specimen MPZ 2023/197 representing a fragmentary carapace valve (‘c1’ in figures 4 and 5).

*Locality and horizon.* Mesones de Isuela (Zaragoza, Spain), *Solenopleuropsis thorali* biozone, Murero Formation, Cambrian: Drumian.

*Description.* The preserved portion of this partial carapace is approximately rectangular (longest axis 10 cm, shortest axis 5 cm), with only part of two margins visible (figure 4). The margin of the carapace is delineated by a thin (*ca* 2 mm) border. The straight margin is interpreted as the dorsal margin. The other margin, which could represent either the anterior or posterior, is curved and can be separated into a process (pr) and an indentation interpreted as a notch (no) (figure 5). The carapace displays a reticulate ornamentation comprised polygons with between four and six sides (‘re’ figure 4). These are smallest close to the margin (*ca* 2 mm diameter) and largest towards the middle of the valve (*ca* 5 mm diameter). Effacement of areas of the carapace means that reticulation is not preserved or clear across the entire surface.

*Remarks.* This fragmentary carapace can be distinguished from the other euarthropod carapaces in the deposit by the thin border along the margin and presence of only a single ornamentation shape, the reticulation. A notch is not reported from the other carapaces but this may be owing to the anterior/posterior parts of many of them being broken, rather than a morphological difference.

The presence of reticulations that increase in size with distance from the margin of the carapace (from *ca* 2 mm to 5 mm), alongside the presence of a notch and process, support assignment of this fragmentary specimen to Tuzoiidae. Large reticulations and cardinal processes which form a notch in the margin of the carapace have been reported from both tuzoiids described to date, *Tuzoia* [54] and *Duplapex* [50]. However other diagnostic features of the family such as dorsal and posterior spines, and a semicircular outline, nor a lateral ridge, cannot be observed from the fragmentary material. Furthermore, large reticulations on a carapace alone are not sufficient to identify this specimen as a tuzoiid, as these are known from a range of Cambrian euarthropods, including hymenocarines, radiodonts and trilobites (e.g. [55]), though the tentative identification of process and notch adjacent to straight dorsal margin makes this assignment most likely. Within tuzoiids, the large size of the carapace is most comparable to material from the Drumian Jince Formation, Czechia [18] which can reach up to 180 mm in length, though specimens of *ca* 120 mm length from the Wuliuan Spence Shale, USA, have also been reported [52]. The fragment of carapace described here exceeds the size of most *Tuzoia* species, which with the exception of the Czech species are no taller than 100 mm ([54] fig. 26). Notably, the Czech *Tuzoia* also display similarly short cardinal processes, creating a small notch in the carapace adjacent to a straight dorsal hinge (e.g. specimen VK41, [18] Plate II B,C).

*Family* Uncertain

**Euarthropod A**

**Figure 6.**
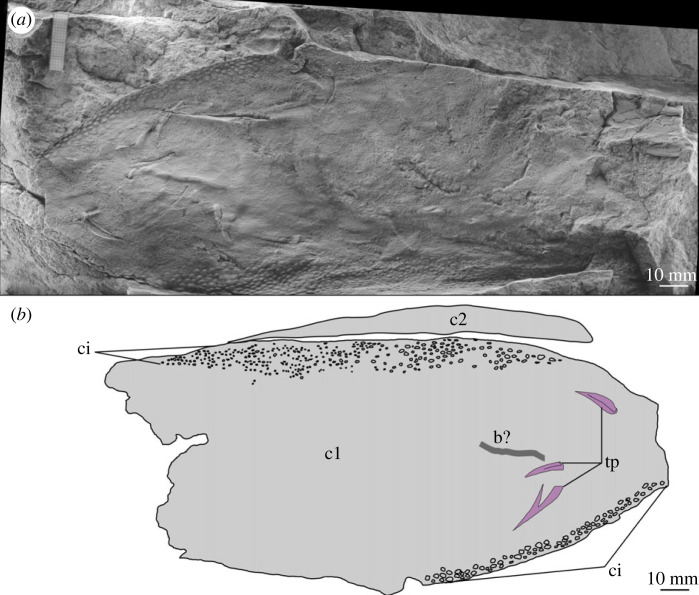
MPZ 2023/194. Paired carapace of euarthropod A from Mesones de Isuela (Zaragoza, Spain), *Solenopleuropsis thorali* biozone, Murero Formation. (*a*) Specimen photograph of latex peel, external view; (*b*) interpretative drawing. b?, possible bioturbation trace; c1, c2; carapace 1 and carapace 2 (paired); ci, elliptical/circular indentation ornament; tp, trilobite pleurae.

**Figure 7.**
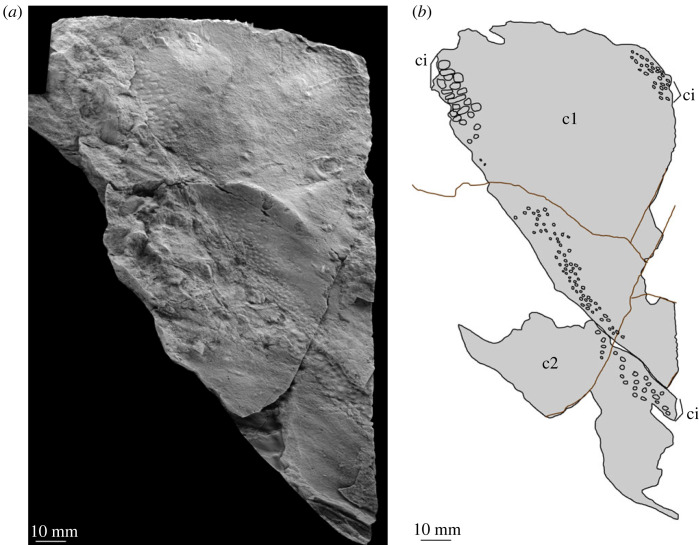
MPZ 2023/198. Paired carapace of euarthropod A from Mesones de Isuela (Zaragoza, Spain), *Solenopleuropsis thorali* biozone, Murero Formation. (*a*) Specimen photograph of latex peel; (*b*) interpretative drawing. c1, c2; carapace 1 and carapace 2 (paired); ci, elliptical/circular indentation ornament.

**Figure 8.**
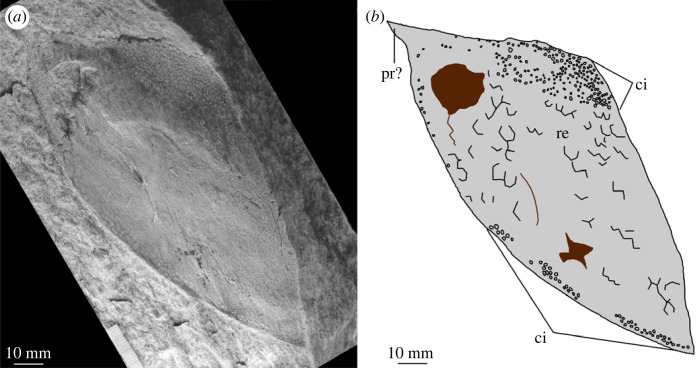
MPZ 2023/195. Carapace of euarthropod A from Mesones de Isuela (Zaragoza, Spain), *Solenopleuropsis thorali* biozone, Murero Formation. (*a*) Specimen photograph of latex peel, external view; (*b*) interpretative drawing. ci, elliptical/circular indentation ornament; pr?, possible process.

**Figure 9.**
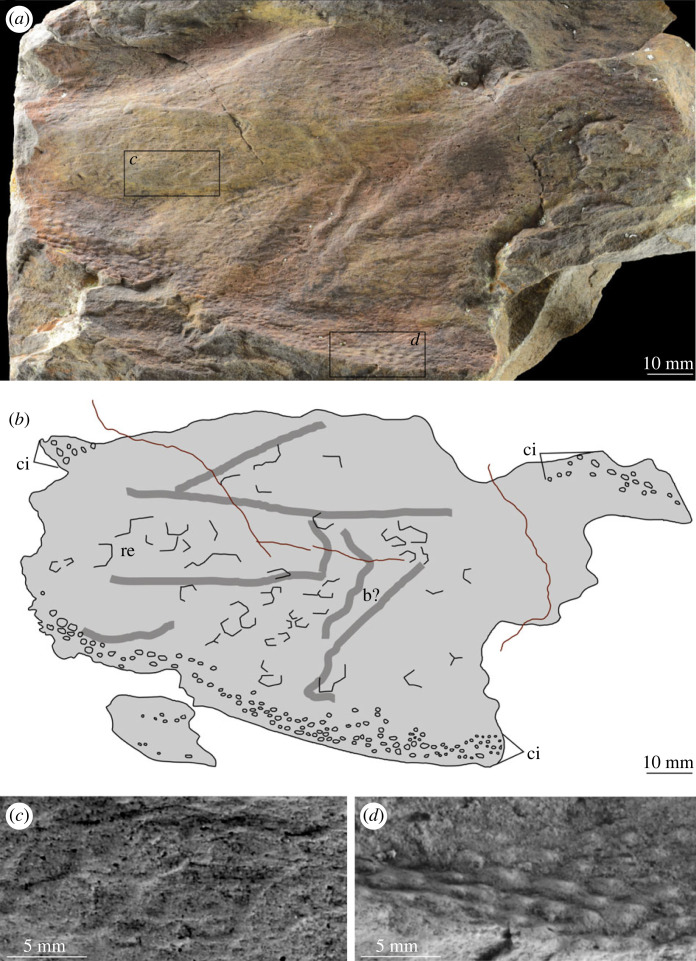
MPZ 2023/196. Carapace of euarthropod A from Mesones de Isuela (Zaragoza, Spain), *Solenopleuropsis thorali* biozone, Murero Formation, preserved in external view. (*a*) Specimen photograph; (*b*) interpretative drawing; (*c*) close up of reticulation ornament; (*d*) close up of elliptical/circular indentation ornament. b?, possible bioturbation trace; ci, elliptical/circular indentation ornament; re, reticulation ornament composed of polygons.

**Figure 10.**
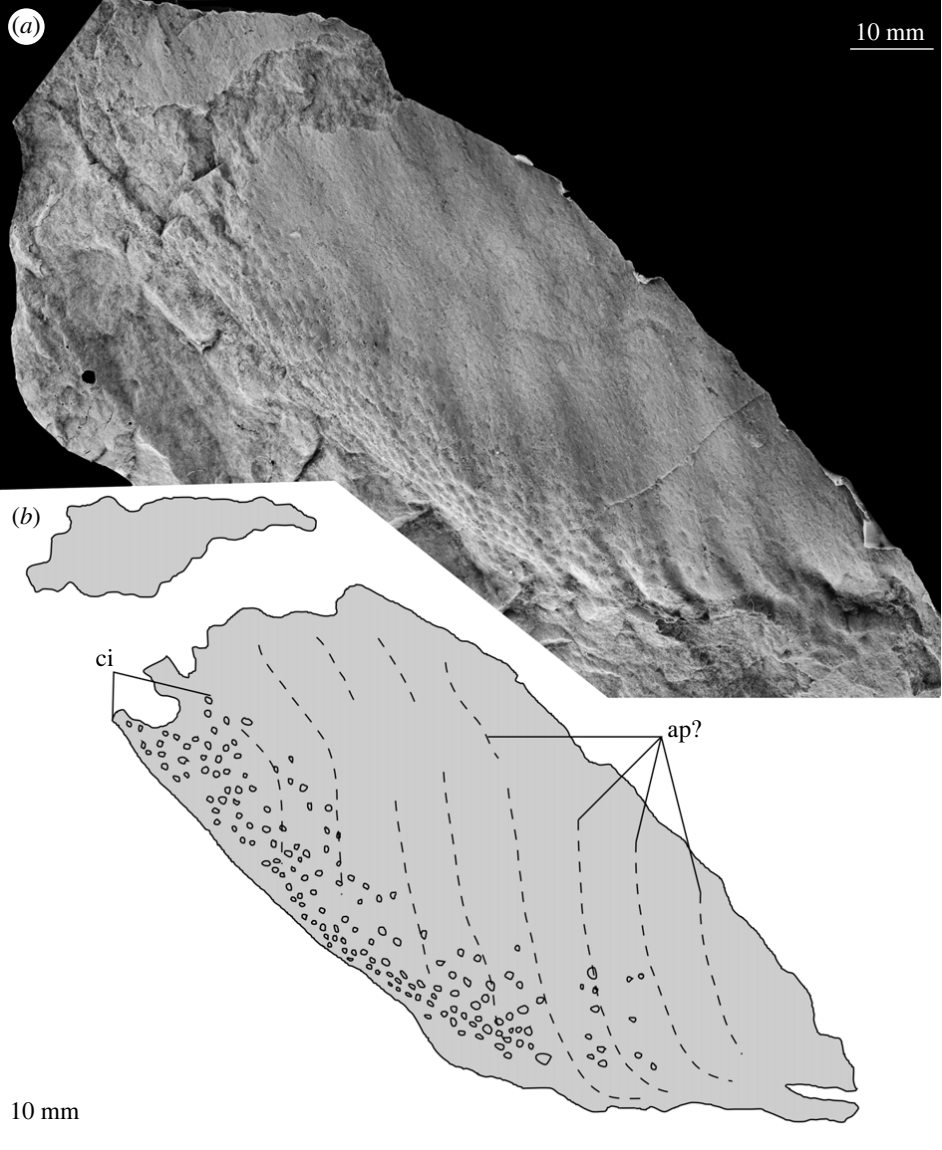
MPZ 2023/192. Carapace and possible appendages of euarthropod A from Mesones de Isuela (Zaragoza, Spain), *Solenopleuropsis thorali* biozone, Murero Formation, preserved in external view. (*a*) Specimen photograph of latex peel; (*b*) interpretative drawing. ap?, possible appendages beneath the carapace; ci, elliptical/circular indentation ornament.

Figure 4 (c2), figures 6–10

*Material.* Six specimens MPZ 2023/192, MPZ 2023/194, MPZ 2023/195, MPZ 2023/196, MPZ 2023/197, MPZ 2023/198 (figures 4 and 6–10), including two paired carapaces (figures 6 and 7), and four partial carapaces (figures 4 and 8–10), one with soft parts beneath (figure 10).

*Locality and horizon.* Mesones de Isuela (Zaragoza, Spain), *Solenopleuropsis thorali* biozone, Murero Formation, Cambrian: Drumian.

*Description.* Partial carapaces range in maximum length parallel to dorsal margin from 62–203 mm, and perpendicular to dorsal margin 70–119 mm (table 1).

**Table 1.** Measurements of size and thickness of ‘circular indentation’ ornament for euarthropod A (Mesones) and ‘large tuzoiid arthropod’ (Jince).

specimen number	figure	max length (parallel to dorsal margin) (mm)	max length (perpendicular to dorsal margin) (mm)	thickness of circular ornament (dorsal) (mm)	thickness of circular ornament (ventral) (mm)
**Mesones**					
MPZ 2023/197	figure 4 (c2)	85	119	9	9
MPZ 2023/194	figure 6	203	88	12	5
MPZ 2023/198	figure 7	133	70	14	10
MPZ 2023/195	figure 8	62	86	15	4
MPZ 2023/196	figure 9	105	76	9^a^	6^a^
MPZ 2023/192	figure 10	n.a.	n.a.	n.a.	11
**Jince**					
L36595	Chlupáč & Kordule [18] fig. 6, p. 173	123	114(227/2)	26	17

^a^Not clear which is dorsal or ventral.

The dorsal margin of carapaces is straight, and in one specimen an articulation can be observed (figure 6). A second specimen displays two valves are preserved in ‘butterfly’ position (figure 7). The ventral margin is curved, and a possible process is preserved in a single specimen (figure 8).

All carapaces display an ornamentation composed of indentations along both dorsal and ventral margins. These elliptical to circular indentations (figure 9*d*) are 1–1.5 mm in diameter, forming bands 4–15 mm in thickness. Ornamentation bands can be up to three times thicker along the dorsal margin than ventral, but in some cases are approximately equal thickness (table 1). In some specimens (figure 4*b* ‘c2’, figures 8 and 9) a second ornamentation can be observed, as a polygonal reticulation (figure 9*c*). Polygons are 4–6 mm in diameter with up to six sides. In multiple specimens raised linear features, representing possible bioturbation, are preserved (‘b?’ in figures 6 and 9*b*). Broken tips of trilobite pleurae are visible beneath one carapace (‘tp’ in figure 6).

A single specimen (figure 10) preserves evidence for soft tissues beneath the carapace. This evidence is provided by eight parallel elongate features of consistent width (5–7 mm), separated by a slight depression in the carapace (‘ap?’, figure 10) which run perpendicular to the ventral margin of the carapace and change direction slightly (angle of 20°) at the same point. These taper to a curved termination close to the ventral margin of the carapace.

*Remarks.* These carapaces can be distinguished from the tuzoiid (above) and euarthropod B (below) by the thickness of the bands of indentations (absent in the tuzoiid, very thin in euarthropod B) and the elongate outline shape of the carapace valves.

The elongate outline, presence of thick band of circular/elliptical indentation ornament, and presence of large *ca* 5 mm reticulations bear strong similarity to the ‘large tuzoiid arthropod’ from the Jince Formation ([18] fig. 6). The Czech specimen falls within the range of sizes described here (table 1), however the thickness of the band of circular/elliptical ornament is wider in the Czech specimen than those described here—both along the dorsal and ventral margins (table 1). The new material described here also preserve features not known in the Czech specimen, including an acuminate termination to one valve (figure 8) and articulating hinge between two valves (figure 6). The presence of an acuminate termination is comparable to the anterior or posterior cardinal processes of *Tuzoia* [54], and could support a tuzoiid affinity for these specimens. However, while some *Tuzoia* specimens show variation in the ornamentation close to the margins of carapace valves (e.g. [54] and [7]) this is expressed as smaller polygons within the broader reticulate pattern (as also shown in figures 4 and 5, above) rather than a distinct ornamentation pattern. Similar ornamentation comprising indents circular to elliptical in outline has been described in a specimen of *Perspicaris? dilatus* from the Rockslide Formation (Drumian: Laurentia [7]), however in this specimen the ornamentation is not limited to the margins as in the Mesones material, but instead covers the whole carapace.

Most notable is the preservation of soft tissues beneath one carapace (figure 10). The close packed nature of the soft tissues, their proximal curvature, and the change in angle at the same distance from the dorsal margin of the carapace indicates that these features probably represent thoracic segments with pleurae or ventral appendages. Comparable features, with ventral appendages extending as far or slightly beyond the carapace margin, have been reported from a range of Cambrian euarthropods with bivalved carapaces from different deposits including *Balhuticaris voltae* (Burgess Shale; [56])*, Clypecaris pteroidea* (Chengjiang; [57]), *Clypecaris serrata* (Xiaoshiba; [58])*, Isoxys curvirostratus* (Chengjiang; [59,60]); *Pauloterminus spinodorsalis* (Sirius Passet; [61]), *Vermontcaris montcalmi* (Parker Quarry; [62]). In *Tuzoia*, appendages do not appear to extend as far as the margin of the carapace (e.g. [51] fig. 3).

Euarthropod A is probably congeneric or conspecific with the ‘large tuzoiid arthropod’ from the Jince Formation ([18] fig. 6, p. 173). The presence of an articulation between the valves indicates that these are indeed large bivalved euarthropods, however there is not strong support for a tuzoiid affinity.

**Euarthropod B**

Figure 11

**Figure 11.**
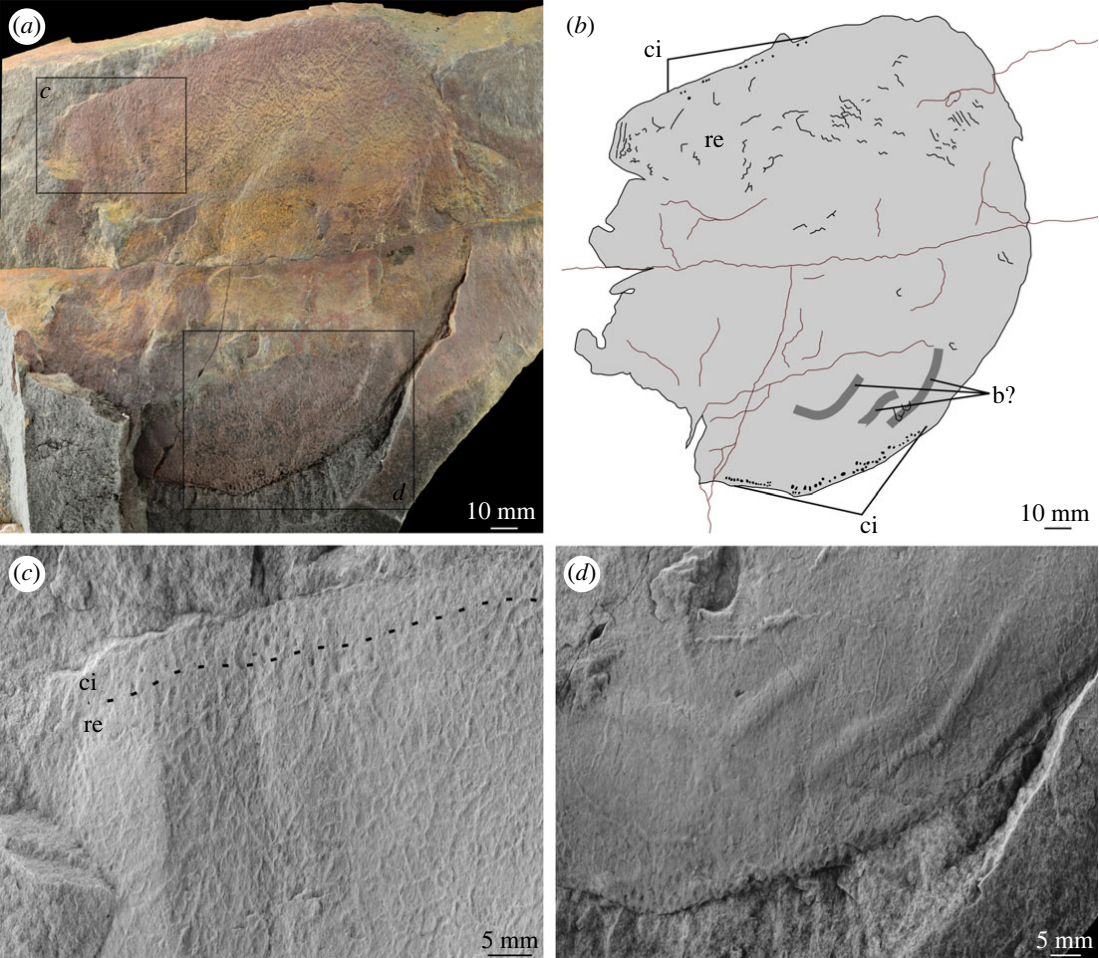
MPZ 2023/193. Euarthropod B from Mesones de Isuela (Zaragoza, Spain), *Solenopleuropsis thorali* biozone, Murero Formation, preserved in external view. (*a*) Specimen photograph; (*b*) interpretative drawing; (*c*) close up of ornamentation; (*d*) close up of possible bioturbation traces. b?, possible bioturbation trace; ci, elliptical/circular indentation ornament; re, reticulation ornament composed of polygons.

*Material.* A single specimen MPZ 2023/193, representing a fragmentary carapace valve (figure 11).

*Locality and horizon*. Mesones de Isuela (Zaragoza, Spain), *Solenopleuropsis thorali* biozone, Murero Formation, Cambrian: Drumian.

*Description.* The preserved portion of this partial carapace is polygonal in outline. The elongate straight margin is interpreted as dorsal. The other margin with two straight sides separated by an obtuse angle is interpreted as ventral. The carapace measures *ca* 180 mm along the longest preserved axis, *ca* 135 mm along the shortest preserved axis. The longest preserved distance parallel to the dorsal margin is 175 mm. The longest preserved distance perpendicular to the dorsal margin is 160 mm.

Two types of ornamentation can be observed. The first of these runs along a thin (*ca* 3–5 mm) band along the margin. This ornamentation is composed of 1 mm diameter circular/elliptical indentations, arranged in rows. Four subparallel rows can be observed along the dorsal margin, with at least three rows along the ventral margin (‘ci’ figure 11*c,d*). The central region of the carapace displays a reticulate polygonal ornamentation, of polygons of between 3–5 sides, around 1.5 mm in diameter (‘re’, figure 11). No clear changes in size or shape can be observed. The central region of the carapace is effaced, and no clear reticulation can be observed in this area.

Three subparallel raised linear features are preserved in the lower half of the carapace. These range between 2.5 and 4 mm wide, and 22–41 mm in length. Two display a change in direction. These are tentatively interpreted as bioturbation traces (‘b?’ figure 11*b,d*).

*Remarks*. This fragmentary carapace can be distinguished from the other carapaces in the deposit by its outline shape (greater distance perpendicular to dorsal margin relative to distance parallel to dorsal margin) and the ornamentation pattern. The presence of two types of ornamentation distinguishes it from the Mesones tuzoiid, while the thin band of circular indentations and smaller size of reticulation polygons distinguishes it from euarthropod A.

The large size of the carapace, presence of reticulation, and straight dorsal margin again draw comparisons between this specimen and tuzoiids. As it is incomplete both anteriorly and posteriorly, it would be larger than any previously described tuzoiid [18,54], however the presence of a second, non-reticulate ornamentation is not known from *Duplaplex* or *Tuzoia* [50,51,54]. Furthermore, the small size of reticulations (*ca* 1.5 mm) compared to the large size of the carapace (greater than 175 mm) is unknown in any *Tuzoia.* Calculated using the incomplete valve length, the Ri value (area of the largest polygon/length of the valve) for this specimen is close to 0.04. This is already at the lower end to the range reported from *Tuzoia* (0.03–0.4; [54]). Indeed a valve length of 235 mm or greater would lead to an Ri value lower than any reported from *Tuzoia*. Notably, the Ri for the Czech *Tuzoia* is close to 0.4 [18,54], an order of magnitude larger than for this specimen. This specimen is also distinguished from the Jince ‘large tuzoiid arthropod’ ([18] fig. 6) by the larger size of reticulations (4–8 mm) and much thicker band of circular indentation ornamentation (up to 26 mm) in the Czech specimen [18].

Other large Cambrian euarthropods with carapaces include *B. voltae, Nereocaris exilis* and *Tegopelte gigas*, and radiodonts such as *Cambroraster, Hurdia, Pahvantia* and *Titanokorys* [56,63–68]. Of these, only radiodont carapace elements display any reticulation or are known to reach approximately the same size as this specimen [55,69]. The carapaces of *B. voltae* and *N. exilis* only cover a small portion of the body and are much smaller than the specimen described here (maximum length *ca* 50 mm; [64] fig. 1; [56] fig. 1), and that of *T. gigas* is ovate and separated into cephalon, thorax and pygidium [63]. However, euarthropod B cannot be confidently identified as a radiodont carapace either, as it does not have a comparable outline shape, nor any notches, spines or lateral extensions that would facilitate detailed comparison to radiodont genera and species [65,66,68–70]. Beyond reticulations, some radiodonts also display marginal rims and linear ornamentations (e.g. *Aegirocassis,* [71]), or tubercles (*Cordaticaris* and *Titanokorys;* [67,70]) but nothing comparable to the distinct band of circular indentations reported here.

Thus, given the unique combination of features that can be observed in this specimen, it is likely that euarthropod B represents a new taxon of large euarthropod—indeed even if all soft parts were covered by the carapace it would still be one of the largest euarthropods as-yet known from the Cambrian. However, given the fragmentary and incomplete nature of the specimen, it is left in open nomenclature pending discovery of additional fossil material.

## 4. Discussion

### 4.1. Do these carapaces represent distinct taxa?

Differences in Palaeozoic euarthropod carapace outline shape, morphology, and ornamentation can be attributed to differences in preservation, including deformation (e.g. [65,72]) decay or effacement [65,73], changes during ontogeny [74], intraspecific variation and dimorphism [74–77], or can be used to diagnose and recognize taxa [50,54,65,78]. In other euarthropods, including large ones such as radiodonts, carapaces are composed of multiple elements with different shapes [10,65–71]. So to what extent can we be confident that the material here belongs to three distinct taxa?

The specimen tentatively assigned to Tuzoiidae, is the most distinct of the three forms identified in that it differs in ornamentation to the other carapaces. The circular indentations are truly absent (rather than not preserved) as the margin of the carapace can be clearly observed. This observation suggests that the tuzoiid does not represent a distinct element of a multipartite carapace belonging to a large radiodont, as lateral and central elements so far described display the same styles of ornamentation, even if it is different between genera (e.g. reticulation in *Cambroraster* and *Hurdia,* [65,68]; puckered and wrinkled texture in *Aegirocassis,* [71]; tubercles in *Cordaticaris* and *Titanokorys,* [67,70]). These differences in ornamentation, in combination with the comparable size of the fragments, can also be marshalled to refute an ontogenetic explanation, as for Cambrian bivalved euarthropods where the ontogeny has been reconstructed, no clear changes in ornamentation have been reported [74,75], although the small sample size of currently known taxa limits the strength of this argument. Dimorphism cannot be ruled out with complete confidence, as bivalved euarthropods where dimorphism has been demonstrated do differ in ornamentation [74,75]. However, dimorphism between the Mesones tuzoiid and euarthropod A or B is deemed unlikely in this case, owing to the greater similarity between euarthropods A and B.

Differences between the specimens assigned to euarthropod A and euarthropod B cannot be ascribed to differences in the level of effacement or deformation, as the thin band of circular/elliptical indentations and start of reticulate pattern can be clearly seen in euarthropod B—precluding the presence of a poorly preserved thicker band more similar to what is observed in euarthropod A. Similarly, the presence of smaller reticulations in euarthropod B cannot be reconciled through deformation, and reflects a biological difference. The presence of a hinge between two valves (figure 6) and comparison with the Jince specimen (L36595; [18] fig. 6, p. 173) indicates that euarthropod A represents a paired carapace, rather than part of a tripartite radiodont carapace. Hinge lines have been reported from a range of bivalved euarthropods, whereas elements in multipartite radiodont carapaces attach anteriorly [65,67]. It is also unlikely that euarthropods A and B represent distinct ontogenetic stages, as the single euarthropod B specimen falls within the distribution of lengths measured from euarthropod A specimens (table 1). However, euarthropod A and B may be dimorphs, rather than distinct taxa, as differences in ornamentation have been demonstrated in other dimorphic Cambrian bivalved euarthropods [74–76], and both display similar ornamentation shapes and styles, differing only in thicknesses of bands and sizes of reticulations.

In summary, the material probably represents at least two, possibly three taxa. A tuzoiid (figures 4 and 5), distinct from euarthropods A and B (figures 6–11). Dimorphism cannot be ruled out for the latter two, which display similar ornamentation patterns (circular/elliptical indents and reticulation) currently distinct from all specimens confidently identified as tuzoiids.

### 4.2. Nature of the possible bioturbation traces

The presence of linear or curved structures beneath some of the carapaces (‘b’ in figures 6, 9, 11) is comparable to features that have been interpreted as burrows and trails beneath other Cambrian bivalved euarthropod carapaces [7,79–83] including from high palaeolatitude Bohemia [84]. It has been shown that complex and high density assemblages of trace fossils with a diverse range of structures were facilitated by bacterially enriched carapaces [79,84]. However, the possible traces reported here are low diversity, represented by a single simple straight to slightly curved type, and low density. In addition, they are only reported from a subset of the carapaces from the deposit. This means that the carapaces from Mesones more likely provided a taphonomic shield for these traces, with the co-occurrence coincidental rather than ecological [79]. Similar traces of comparable age from Laurentia, also occurring beneath only some of the many carapaces recovered [7,80], were interpreted as empty tubes occurring post-mortem [7].

### 4.3. Did bivalved euarthropods have larger body sizes at higher latitudes in the Cambrian?

Even with the limited amount of material from higher latitude deposits preserving non-biomineralized tissues studied so far (e.g. [18], this study), it is evident that they are important for understanding the diversity and geographical distribution of Cambrian animals. Cambrian Lagerstätten assemblages from low latitudes have been shown to become more homogeneous through time [85]. However, the bivalved euarthropods from higher latitudes of Gondwana—including those described herein—are distinct from coeval taxa from equatorial and tropical palaeolatitudes. This demonstrates the importance of taking a global approach to reconstructing past diversity, as higher latitude deposits are—unsurprisingly—revealing taxa not known from lower latitude sites and thus increasing the total known biodiversity from this time.

Coeval deposits covering a range of latitudes are also necessary for determining palaeogeographic patterns. For example, the large tuzoiid (alongside material described by [18,24]) provides evidence that the family Tuzoiidae had a broad biogeographic range during the middle Cambrian. The large size of all the euarthropod carapaces in the study compared to lower latitude bivalved euarthropods—the tuzoiids are the largest known in the family [54], while euarthropods A and B are even larger—indicate that euarthropods may have been larger at higher latitudes during the Drumian.

Larger size in members of a clade from high latitudes—an ecographical observation often referred to as Bergmann's Rule—has been reported in extant ectotherms including marine euarthropods [86–89]. In the light of this research, the larger size of bivalved euarthropods at high latitudes in the Cambrian could relate to a range of factors, including differences in temperature and subsequent growth rates and oxygen availability, primary productivity and/or predation pressure. For larger marine animals, such as the euarthropods described herein, temperature-driven oxygen availability is thought to be particularly important [86,87,90]. The narrow profile and relatively low surface-area to volume ratio of Cambrian bivalved euarthropods makes this a plausible explanation for size variation with latitude within the group. Indeed, while the early Cambrian was probably a greenhouse world, sea surface temperatures at the poles have been modelled to have been 20°C cooler than close to the Equator (15°C compared to 35°C at the Equator) [91]. In the modern ocean the temperature difference is larger (*ca* 0°C at high latitudes, *ca* 25–30°C at the Equator) [91].

Larger sizes at high latitudes may not have only been limited to bivalved euarthropods. The radiodont *Caryosyntrips,* a lower-stem group euarthropod described from the older (Stage 4) Valdemiedes Formation, was also much larger than its Burgess Shale and Great Basin counterparts [10,38,92,93]. Furthermore, the two palaeoscolecids figured (figure 3) from the same levels as euarthropod carapaces measure 206 mm in length and 12 mm wide in one case, and 90 mm in length (incomplete) and 9 mm in width in the other. These sizes are in the range of other palaeoscolecids described from high latitude deposits [94], and larger than low latitude representatives. As our size-latitude observations are currently limited to only a few high-latitude sites, and do not consider other variables (e.g. water depth, environment, nutrient availability), these results are only preliminary at this stage.

## 5. Conclusion

Euarthropod carapaces of three distinct morphologies, representing at least two taxa, are the first non-biomineralized fossils reported from a deposit near Mesones de Isuela (Cambrian: Drumian, Spain). This deposit is unusual for Drumian deposits of this kind, as it records a relatively high latitude—most Drumian deposits preserving non biomineralized fossils fall close to the palaeo-equator.

High latitude deposits preserving non-biomineralized tissues and animals provide an important window into the diversity and palaeogeography of the Cambrian. Indeed the most abundant carapace morph (euarthropod A) from Mesones has not been reported from any of the numerous coeval low latitude deposits, while euarthropod B is currently only known from this deposit. Our limited data thus far indicates that Cambrian bivalved euarthropods living at higher latitude may have been larger than those from low latitudes.

## Data Availability

Specimens are accessioned at Museo de Ciencias Naturales de la Universidad de Zaragoza (MPZ).
